# Oligogenic structure of amyotrophic lateral sclerosis has genetic testing, counselling and therapeutic implications

**DOI:** 10.1136/jnnp-2024-335364

**Published:** 2025-02-13

**Authors:** Alfredo Iacoangeli, Allison A Dilliott, Ahmad Al Khleifat, Peter M Andersen, Nazlı A Başak, Johnathan Cooper-Knock, Philippe Corcia, Philippe Couratier, Mamede deCarvalho, Vivian E Drory, Jonathan D Glass, Marc Gotkine, Yosef M Lerner, Orla Hardiman, John E Landers, Russell L McLaughlin, Jesus S Mora Pardina, Karen Morrison, Susana Pinto, Monica Povedano, Christopher E Shaw, Pamela J Shaw, Vincenzo Silani, Nicola Ticozzi, Philip van Damme, Leonard H van den Berg, Patrick Vourc'h, Markus Weber, Jan Herman Veldink, Project MinE ALS Sequencing Consortium, Richard Dobson, Guy A Rouleau, Ammar Al-Chalabi, Sali M K Farhan

**Affiliations:** 1 Department of Biostatistics and Health Informatics, King's College London, London, UK; 2 Department of Basic and Clinical Neuroscience, King's College London, London, UK; 3 Perron Institute for Neurological and Translational Science, Perth, Western Australia, Australia; 4 Biomedical Research Centre, South London and Maudsley NHS Foundation Trust, London, UK; 5 Department of Neurology and Neurosurgery, McGill University, Montreal, Quebec, Canada; 6 Montreal Neurological Institute-Hospital, McGill University, Montreal, Quebec, Canada; 7 Clinical Science, Neurosciences, Umeå Universitet Medicinska Fakulteten, Umea, Sweden; 8 Suna and İnan Kıraç Foundation, Neurodegeneration Research Laboratory (NDAL), KUTTAM, Koç University School of Medicine, Istanbul, Turkey; 9 Sheffield Institute for Translational Neuroscience (SITraN), University of Sheffield, Sheffield, UK; 10 UMR 1253, Université de Tours, Inserm, Tours, France; 11 Centre de référence sur la SLA, CHU de Tours, Tours, France; 12 Centre de référence sur la SLA, CHRU de Limoges, Limoges, France; 13 UMR 1094, Université de Limoges, Inserm, Limoges, France; 14 Instituto de Fisiologia, Universidade de Lisboa, Lisbon, Portugal; 15 Department of Neurology, Sourasky Medical Centre, TelAviv, Israel; 16 Faculty of Medicine, Tel-Aviv University, Tel-Aviv, Israel; 17 Neurology, Emory University, Atlanta, Georgia, USA; 18 Faculty of Medicine, Hebrew University of Jerusalem, Jerusalem, Israel; 19 Department of Neurology, Hadassah Medical Center, Jerusalem, Israel; 20 Academic Unit of Neurology, Trinity College Dublin, Dublin, Ireland; 21 Department of Neurology, University of Massachusetts Medical School, Worcester, Massachusetts, USA; 22 Smurfit Institute of Genetics, Trinity College Dublin, Dublin, Ireland; 23 ALS Unit, San Rafael Hospital, Madrid, Spain; 24 School of Medicine, Dentistry & Biomedical Sciences, Queen's University Belfast, Belfast, UK; 25 Functional Unit of Amyotrophic Lateral Sclerosis (UFELA), Hospital de Bellvitge, L'Hospitalet de Llobregat, Barcelona, Spain; 26 Department of Pathophysiology and Transplantation, University of Milan, Milano, Italy; 27 Department of Neurology and Laboratory of Neuroscience, IRCCS Istituto Auxologico Italiano, Milan, Italy; 28 Department of Neuroscience, Leuven Brain Institute (LBI), VIB, Center for Brain and Disease Research, University of Leuven, Leuven, Belgium; 29 Department of Neurology, University Hospitals Leuven, Leuven, Belgium; 30 Department of Neurology, University Medical Centre Utrecht Brain Centre, Utrecht, Netherlands; 31 Service de Biochimie et Biologie molécularie, CHU de Tours, Tours, France; 32 Neuromuscular Diseases Unit, Kantonsspital St. Gallen, St. Gallen, Switzerland; 33 Department of Genetics, McGill University, Montreal, Quebec, Canada; 34 Department of Neurology, King’s College Hospital, London, UK

**Keywords:** ALS, GENETICS, MOTOR NEURON DISEASE

## Abstract

**Background:**

Despite several studies suggesting a potential oligogenic risk model in amyotrophic lateral sclerosis (ALS), case–control statistical evidence implicating oligogenicity with disease risk or clinical outcomes is limited. Considering its direct clinical and therapeutic implications, we aim to perform a large-scale robust investigation of oligogenicity in ALS risk and in the disease clinical course.

**Methods:**

We leveraged Project MinE genome sequencing datasets (6711 cases and 2391 controls) to identify associations between oligogenicity in known ALS genes and disease risk, as well as clinical outcomes.

**Results:**

In both the discovery and replication cohorts, we observed that the risk imparted from carrying multiple ALS rare variants was significantly greater than the risk associated with carrying only a single rare variant, both in the presence and absence of variants in the most well-established ALS genes. However, in contrast to risk, the relationships between oligogenicity and ALS clinical outcomes, such as age of onset and survival, did not follow the same pattern.

**Conclusions:**

Our findings represent the first large-scale, case–control assessment of oligogenicity in ALS and show that oligogenic events involving known ALS risk genes are relevant for disease risk in ~6% of ALS but not necessarily for disease onset and survival. This must be considered in genetic counselling and testing by ensuring to use comprehensive gene panels even when a pathogenic variant has already been identified. Moreover, in the age of stratified medication and gene therapy, it supports the need for a complete genetic profile for the correct choice of therapy in all ALS patients.

**WHAT IS ALREADY KNOWN ON THIS TOPIC**

- Although case-level evidence has suggested an oligogenic risk model in amyotrophic lateral sclerosis (ALS), case–control statistical evidence implicating oligogenicity with disease risk or clinical outcomes is limited.

**WHAT THIS STUDY ADDS**

- We have provided robust evidence that carrying multiple ALS rare variants leads to greater ALS risk than carrying only one, while the relationships between oligogenicity and age of onset and survival, did not follow the same pattern.

**HOW THIS STUDY MIGHT AFFECT RESEARCH, PRACTICE OR POLICY**

- For all ALS patients, comprehensive gene panels should be used in genetic testing and counselling, and a complete genetic profile should be used for the correct choice of therapy, even when a pathogenic variant has already been identified.

## Introduction

 As an intermediary between monogenic and polygenic disease transmission, oligogenic inheritance refers to the additive moderate phenotypic effect of genetic variants in a few genes that together drive disease presentation. Establishing whether oligogenicity plays a role in the development of a disease is important for more accurate diagnosis, as it may clarify the role of low penetrance variants or explain atypical clinical presentations. It could also drive the development of treatment by highlighting multiple potential targets. Therefore, identifying an oligogenic component of risk in a disease considered to be predominantly monogenic has direct implication for genetic counselling and risk assessment.^1^ Although not well studied, oligogenicity has been described in a selected number of diseases such as Bardet-Biedl syndrome, Charcot-Marie-Tooth and long QT syndrome.^2^,^8^ Interestingly, all three forms of transmission—monogenic, polygenic and oligogenic—have been reported in amyotrophic lateral sclerosis (ALS).

In recent years, large-scale case–control analyses have been prioritised in the study of monogenic and polygenic forms of ALS, often including thousands of samples to maximise the statistical power of discovery.^9^,^16^ However, the same effort has not been put forward to investigate oligogenic events driving ALS risk. Case-level evidence has suggested an oligogenic risk model in ALS,^17 18^ which conforms with the proposed multistep hypothesis of ALS that describes multiple molecular events—including the possibility of multiple genetic variants—occurring to trigger ALS onset.^19 20^ Yet large case–control analyses have not been performed using ALS cohorts to confirm the role of oligogenicity in the disease. Previous reports of the phenomenon had modest sample sizes or were limited to case studies that did not include control cohorts and did not directly assess the disease risk or influence on clinical outcomes imparted by carrying multiple variants in ALS genes with respect to carrying only one.^1718 21^,^27^ As a result, although current evidence suggests an effect of oligogenic events on disease risk, statistically robust evidence is not available, and it is not clear whether such genetic events can act as disease modifiers.

In order to fill this gap, we leveraged the Project MinE ALS Sequencing Consortium large-scale, genome sequencing datasets of individuals with ALS (n=6711) and controls (n=2391) to identify signals of association between oligogenicity in known ALS genes and disease risk. Further, we investigated whether carrying multiple rare variants influences clinical features of disease, including age of onset and survival. The presented analyses represent the first large-scale, case–control assessment of oligogenic associations in ALS to date.

## Methods

### Study cohort and sample sequencing

Genome sequencing data obtained from the Project MinE ALS Sequencing Consortium were composed of a discovery subset (individuals with ALS=4518 and controls=1821) and a replication cohort (individuals with ALS=2193 and controls=570) corresponding to the two main releases of Project MinE (data freeze 1 and 2). Details regarding sequencing methodology and quality control were previously described.^28^,^30^ Briefly, all samples were sequenced using either the Illumina HiSeq 2000 platform or Illumina HighSeq X platform (San Diego, California, USA), and sequencing reads were aligned to the hg19 reference genome to call single nucleotide variants, insertions and deletions. Quality control was performed at both an individual and variant level and included assessment of read depth and coverage, ancestry-defining principal component analysis (PCA) and identity-by-descent analysis. Controls were matched to the individuals with ALS based on age, sex and geographical region.

Post quality control, 4299 individuals with ALS and 1815 controls from the Project MinE discovery subset, and 2057 individuals with ALS and 513 controls from the replication cohort were retained for analysis. Genetic data and clinical outcomes, including age of ALS onset and survival period, were available for the ALS patients. Survival period was defined as years from diagnosis to death, or years from diagnosis to last follow-up, as appropriate.

### Variant annotation and filtering

Variants were annotated using the Ensembl variant effect predictor and Annovar.^31 32^ Variants were further annotated based on minor allele frequencies (MAFs) using the Genome Aggregation Database (gnomAD) V.2.1.1 non-neurological dataset.^33^ Only variants considered rare, with an MAF<0.01 in both gnomAD and the Project MinE controls, were retained for further analysis.

26 genes previously associated with ALS in the literature were selected for the analysis, as previously described,^34^ and are hereafter referred to as known ALS genes (online supplemental table 1). Only genes with at least two publications reporting rare coding variants in individuals with ALS were included. Genes with unclear inheritance patterns or limited or refuted gene-disease validity classification from the ClinGen ALS Gene Curation Expert Panel (GCEP) as of January 2022 were excluded (Clinical Genome ALS). Genes were also subclassified based on the strength of evidence regarding their association with ALS. ‘Established ALS genes’ included those with a definitive relationship with ALS based on curation by the ClinGen ALS GCEP. All other genes were considered ‘ALS-associated genes’. For *C9orf72* and *ATXN2,* only the ALS-associated repeat expansions were included, while for the remaining 24 known ALS genes only variants within the protein coding regions were retained.

The rare, protein-coding variants were binned into three classes: (1) synonymous variants; (2) missense variants (missense single nucleotide variants and in-frame insertions or deletions) and (3) protein truncating variants (PTVs; stop lost and gained, start lost, transcript amplification, frameshift, transcript ablation, and splice acceptor and donor variants). Missense variants were also annotated using REVEL (>0.75)^35^ and AlphaMissense (classification of ‘likely pathogenic’)^36^ to identify missense variants predicted to be pathogenic.

### Pathogenic repeat expansion detection

All genome samples were also assessed for the *C9orf72* GGGGCC and *ATXN2* CAG repeat expansions using ExpansionHunter.^37^ ExpansionHunter has been previously validated for the repeat expansions in both *C9orf72* and *ATXN2*.^38^,^40^ A hexanucleotide repeat expansion of >30 copies in *C9orf72* is considered pathogenic for ALS, whereas a trinucleotide intermediate repeat expansion of 29–32 copies in *ATXN2* is considered a risk factor for ALS.^41^,^43^

### Statistical analyses

To model the influence of carrying a single variant in a known ALS gene (singleton) or carrying at least one variant in more than one known ALS genes (oligogenic) on ALS risk and clinical outcomes, regression analyses were applied. More specifically, risk of ALS as a function of singleton or oligogenic carrier status was assessed in individuals with ALS and controls using logistic regressions adjusting for sex, 10 ancestry-defining PCs and total genetic load (summation of synonymous, missense and PTVs, as previously described).^9^ Associations between ALS age of onset and singleton or oligogenic carrier status in individuals with ALS were assessed using linear regressions adjusting for sex, site of onset, 10 ancestry-defining PCs and total genetic load. Finally, associations between ALS survival period and singleton or oligogenic carrier status in individuals with ALS were assessed using Cox proportional hazard regressions adjusting for the same covariates described above.

To control for any residual synonymous inflation not fully corrected by population structure-based PCs, and to determine whether the *C9orf72* or *ATXN2* repeat expansions were driving associations, all regression models assessed associations with five variant type bins: (1) synonymous variants; (2) missense variants and PTVs; (3) missense variants, PTVs and *ATXN2* repeats; (4) missense variants, PTVs and *C9orf72* repeats and (5) missense variants, PTVs, *ATXN2* repeats and *C9orf72* repeats. Statistical analyses were performed using the *logistf* library from R statistical software (V.4.2.2)^44^ in R Studio (V.1.1.414). Data visualisation was performed using the *ggplot2* R package (V.3.3.6).^45^

## Results

### Oligogenic risk of ALS

Following the assessment for rare single nucleotide variants and repeat expansions in known ALS genes, we determined the number of singleton and oligogenic carriers (table 1). In total, 1161 individuals with ALS (n=4299) were considered singleton carriers, defined as carrying one non-synonymous, rare variant in one known ALS gene, in the Project MinE discovery subset. Whereas 255 individuals with ALS were considered oligogenic carriers, defined as carrying at least one non-synonymous, rare variant in two or more known ALS genes, in the Project MinE discovery subset. Similarly, 540 individuals with ALS (n=2057) and 131 individuals with ALS were considered singleton and oligogenic carriers, respectively, in the Project MinE replication subset. We attempted to further stratify variants by focusing on missense variants predicted to be pathogenic by either REVEL or AlphaMissense and by considering PTVs alone. Only 19 of 4299 people with ALS and 5 of 1815 controls were oligogenic carriers of such variants. Given the extremely low frequency of oligogenicity involving such rare variants, and the consequential limited statistical power provided by our sample to test their association with disease risk, we decided to include all non-synonymous variants in our analyses.

**Table 1:** Age of onset and survival based on the number and type of rare variants (MAF<0.01) carried by individuals with ALS from the Project MinE ALS Sequencing Consortium

Variant type	ALS carriers (n=4299)	Age of onset (years)			Survival (years)		
		Mean	Median	SD	Mean	Median	SD
No variants	2883	62.67	63.57	11.71	3.42	2.71	2.65
Singleton	1161	61.60	62.65	11.50	3.43	2.70	2.71
Oligogenic (All ALS genes)	255	61.83	62.90	10.93	3.47	2.71	3.02
Oligogenic (Established ALS genes)	129	60.85	61.57	10.63	3.68	2.87	3.09
Oligogenic (ALS-associated genes)	31	62.24	65.70	11.90	2.93	2.25	2.16
Oligogenic (≥1 variant in established ALS gene and ≥1 variant in ALS-associated Gene)	95	63.41	64.94	10.93	3.49	2.65	3.28

ALS, amyotrophic lateral sclerosis; MAF, minor allele frequency.

MAFs were obtained from the GnomAD V.2.1.1 non-neurological dataset. Singleton refers to carrying one variant in one ALS gene. Oligogenic refers to carrying at least one variant in ≥2 ALS genes. Established ALS genes include those with a definitive relationship with ALS based on curation by the ClinGen ALS GCEP. All other genes were considered ALS-associated genes.

**Figure 1:**
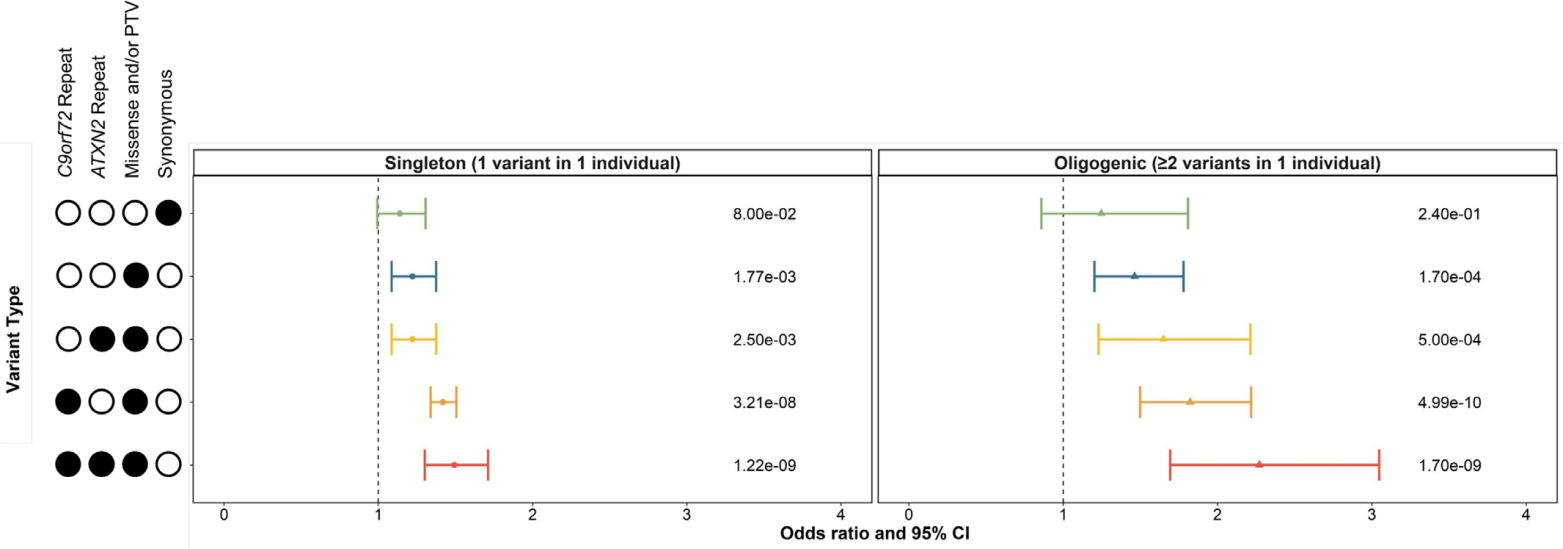
Singleton and oligogenic enrichment of rare variants (MAF < 0.01) in known ALS genes in individuals with ALS compared to controls. Enrichment of carriers of rare variants in one known ALS gene and carriers of rare variants in two or more known ALS genes was compared to non-carriers in individuals with ALS compared to controls. Enrichment analyses were performed using logistic regression in the discovery subset (individuals with ALS = 4299, controls = 1815) of the Project MinE ALS sequencing consortium dataset, including sex, 10 ancestry defining principal components, and total variant count (total genetic load) as covariates. The upset plot on the left legend indicates the variant types that the singleton or oligogenic variant may encompass; for example, the bottom row indicates the singleton variant or two or more oligogenic variants may be a C9orf72 repeat expansion, ATXN2 repeat expansion, missense variants, or protein truncating variants (PTVs). Synonymous variants, missense variants, and PTVs were identified in 24 known ALS genes using whole genome sequencing. ATXN2 and C9orf72 refer to a pathogenic repeat expansion being identified in the respective gene using ExpansionHunter. Minor allele frequencies were obtained from the GnomAD v2.1.1 non-neurological dataset. Abbreviations: CI, confidence interval; MAF, minor allele frequency; PTV, protein truncating variant.

Using our model, an enrichment analysis indeed demonstrated that carrying ≥2 rare (MAF<0.01), non-synonymous variants in multiple ALS genes was significantly associated with disease risk, with greater odds than observed in the singleton analysis (figure 1). The increased odds relative to singleton carrier status were observed for oligogenic carriers when only missense variants or PTVs were considered (singleton OR=1.22 (95% CI 1.09 to 1.37), p=1.77e−03; oligogenic OR=1.46 (95% CI 1.20 to 1.78), p=1.70e−04), as well as when *C9orf72* repeat expansions (singleton OR=1.42 (95% CI 1.34 to 1.51), p=3.21e−08; oligogenic OR=1.82 (95% CI 1.50 to 2.22), p=4.99e−10), *ATXN2* repeat expansions (singleton OR=1.22 (95% CI 1.09 to 1.37), p=2.50e−03; oligogenic OR=1.65 (95% CI 1.23 to 2.21), p=5.00e−04) and both repeat expansions (singleton OR=1.49 (95% CI 1.30 to 1.71), p=1.22e−09; oligogenic OR=2.27 (95% CI 1.69 to 3.05), p=1.70e−09) were included in the analysis.

On confirmation of our risk analysis using the Project MinE replication subset, we observed that singleton carrier status was only significantly associated with ALS when missense variants or PTVs in known ALS genes and the *C9orf72* and *ATXN2* repeat expansions were included in the analysis (OR=1.30 (95% CI 1.05 to 1.61), p=1.70e–02; online supplemental figure 1). In contrast, oligogenic carrier status was significantly associated with ALS when only missense variants or PTVs were considered (OR=1.39 (95% CI 1.02 to 1.90), p=4.00e–02), as well as when *C9orf72* repeat expansions (OR=1.57 (95% CI 1.15 to 2.15), p=4.50e–03), *ATXN2* repeat expansions (OR=1.49 (95% CI 1.07 to 2.08), p=1.30e–02) and both repeat expansions (OR=1.73 (95% CI 1.24 to 2.42), p=5.70e–04) were included in the analysis.

We also assessed whether oligogenic carrier status of variants with lower MAFs was associated with an increased risk of ALS in comparison to singleton carrier status. In a similar manner to the MAF<0.01 rare variant assessment, oligogenic carrier status for variants of MAF<0.001, MAF<0.0001 and AC=1 were significantly associated with disease risk, with greater odds than observed in the singleton analyses of the Project MinE discovery subset (online supplemental figure 2). These findings were replicated in the analyses of singleton and oligogenic carrier statuses of variants with lower MAFs in the Project MinE replication cohort (online supplemental figure 1).

### Oligogenic influence on ALS clinical outcomes

From the Project MinE discovery cohort, 4299 individuals with ALS had their age of onset and survival periods captured. Summary of the clinical outcomes of individuals with ALS carrying zero, singleton or oligogenic non-synonymous, rare variants in known ALS genes are presented in table 1.

**Figure 2:**
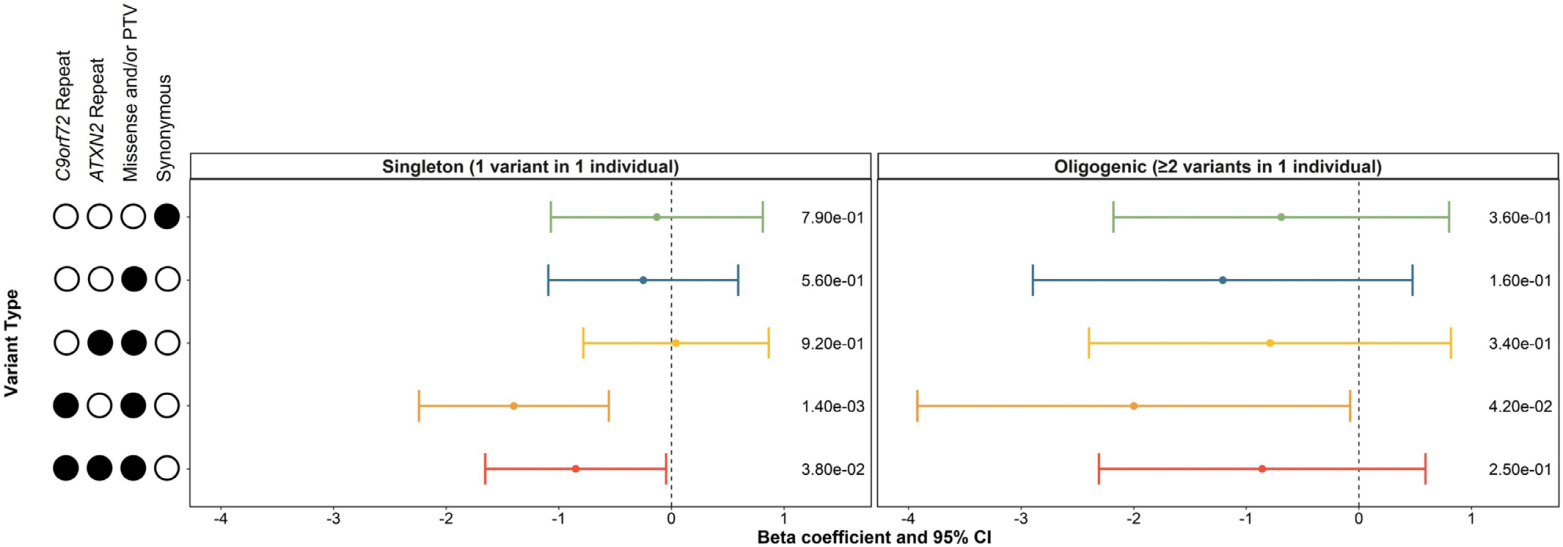
Influence of singleton and oligogenic enrichment of rare variants (MAF < 0.01) in known ALS genes to ALS age of onset. The influence of carrying a rare variant in a single known ALS genes was compared to the influence of carrying rare variants in two or more known ALS genes on ALS age of onset in the discovery cohort of the Project MinE ALS sequencing consortium (individuals with ALS = 4299). Enrichment analyses were performed using linear regression including sex, site of onset, 10 ancestry defining principal components, and total variant count (total genetic load) as covariates. The upset plot on the left legend indicates the variant types that the singleton or oligogenic variant may encompass; for example, the bottom row indicates the singleton variant or two or more oligogenic variants may be a C9orf72 repeat expansion, ATXN2 repeat expansion, missense variants, or protein truncating variants (PTVs). Synonymous variants, missense variants, and PTVs were identified in 24 known ALS genes using whole genome sequencing. ATXN2 and C9orf72 refer to a pathogenic repeat expansion being identified in the respective gene using ExpansionHunter. Minor allele frequencies were obtained from the GnomAD v2.1.1 non-neurological dataset. Abbreviations: CI, confidence interval; MAF, minor allele frequency; PTV, protein truncating variant.

Linear regressions adjusting for sex, site of onset, 10 ancestry-defining PCs and total variant count were applied to determine whether singleton or oligogenic carrier statuses were associated with ALS age of onset. We found that carrying a singleton, rare (MAF<0.01), non-synonymous variant was significantly associated with lower age of onset when the *C9orf72* repeat expansion was included in the analysis (β=−1.40 (95% CI −2.24 to −0.56), p=1.4e−03; figure 2). The singleton association was also observed for variants with an MAF<0.001 and MAF<0.0001 when the *C9orf72* repeat expansion was included in the analyses and for MAF<0.0001 when the *C9orf72* repeat expansion was excluded (p=0.031) (online supplemental figure 3).

Carrying oligogenic, rare (MAF<0.01), non-synonymous variants in multiple known ALS genes was only marginally significantly associated with age of onset when the *C9orf72* repeat expansion was included in the analysis (p=0.042, figure 2), while a lack of oligogenic association was observed for variants of lower MAF (online supplemental figure 3).

**Figure 3:**
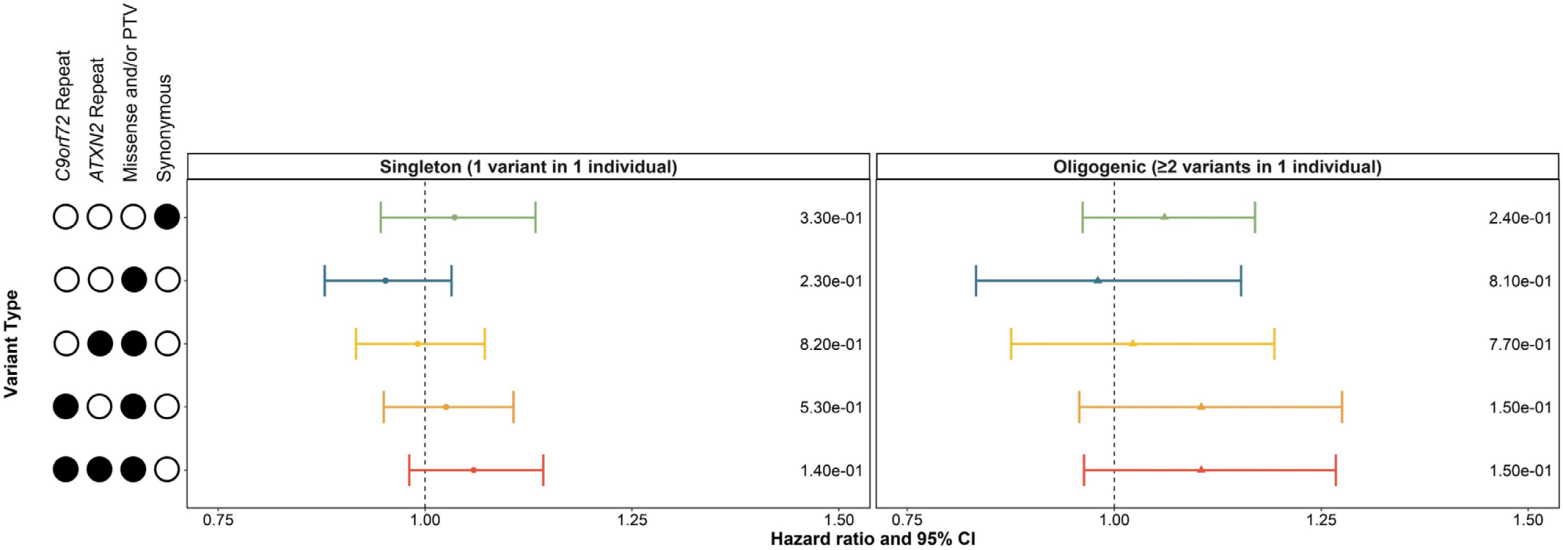
Influence of singleton and oligogenic enrichment of rare variants (MAF < 0.01) in known ALS genes to ALS survival period. The influence of carrying a rare variant in a single known ALS gene was compared to the influence of carrying rare variants in two or more known ALS genes on ALS survival period in the discovery cohort of the Project MinE ALS sequencing consortium (individuals with ALS = 4299). Enrichment analyses were performed using Cox proportional-hazards models including sex, site of onset, 10 ancestry defining principal components, and total variant count (total genetic load) as covariates. The upset plot on the left legend indicates the variant types that the singleton or oligogenic variant may encompass; for example, the bottom row indicates the singleton variant or two or more oligogenic variants may be a C9orf72 repeat expansion, ATXN2 repeat expansion, missense variants, or protein truncating variants (PTVs). Synonymous variants, missense variants, and PTVs were identified in 24 known ALS genes using whole genome sequencing. ATXN2 and C9orf72 refer to a pathogenic repeat expansion being identified in the respective gene using ExpansionHunter. Minor allele frequencies were obtained from the GnomAD v2.1.1 non-neurological dataset. Survival period was defined as years from diagnosis to death, or years from diagnosis to last follow-up, as appropriate. Abbreviations: CI, confidence interval; MAF, minor allele frequency; PTV, protein truncating variant.

Similarly, Cox proportional hazard models adjusting for sex, site of onset, 10 ancestry-defining PCs and total variant count were applied to determine whether singleton or oligogenic carrier statuses were associated with ALS survival period. We found that carrying a singleton, rare (MAF<0.01), non-synonymous variant was not significantly associated with ALS survival period (figure 3); however, carrying a singleton missense variant or PTV of MAF<0.001 and MAF<0.0001, or a *C9orf72* repeat expansion were significantly associated with an increased HR (p_0.001_=0.041 and p_0.0001_=0.0056, online supplemental figure 4).

Carrying oligogenic, rare, non-synonymous variants in multiple known ALS genes was not associated with decreased or increased survival period, for any MAF classes (online supplemental figure 4).

### Gene involvement in oligogenic events

**Figure 4:**
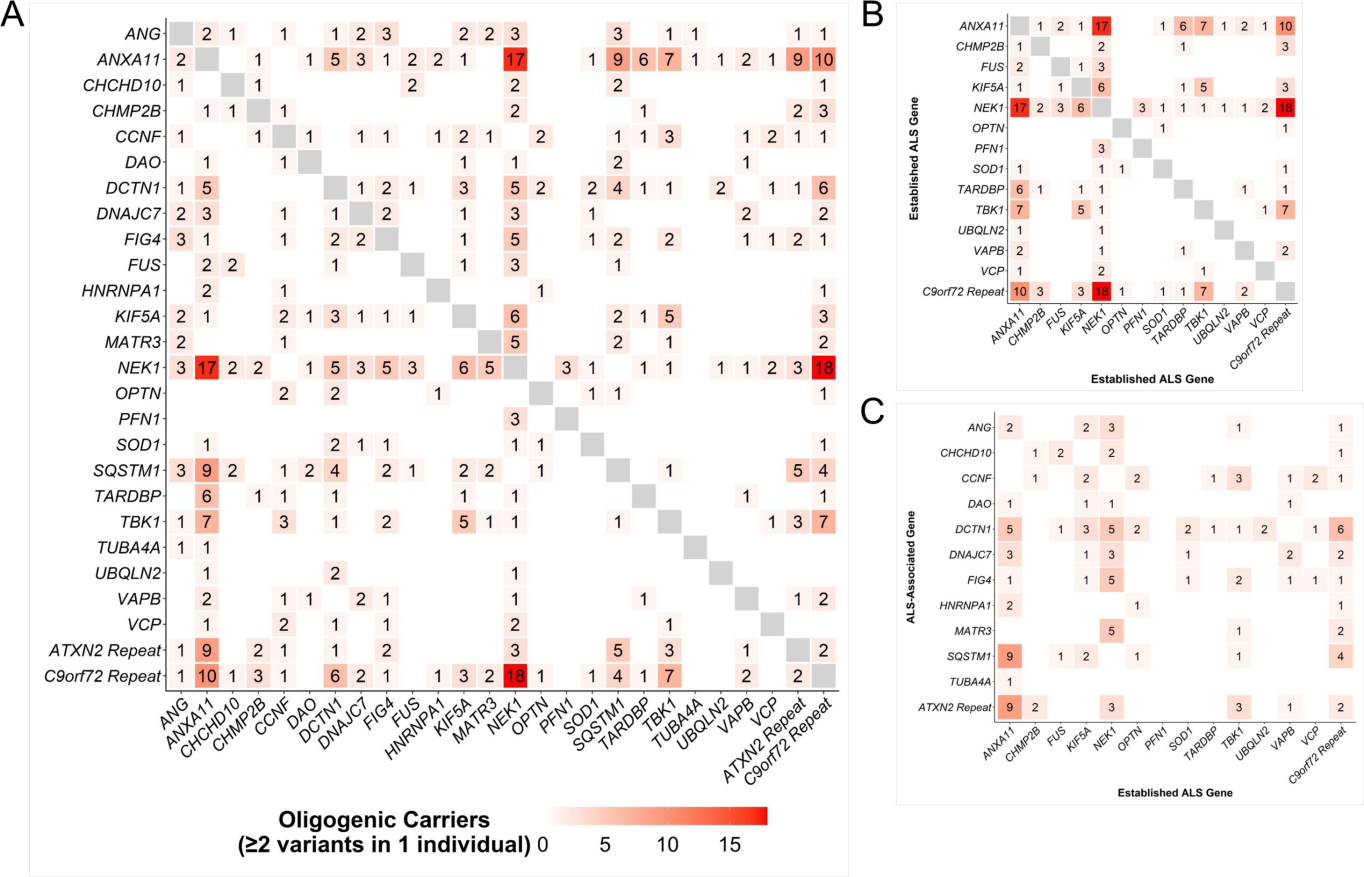
Individuals with ALS carrying two or more rare variants in ALS genes. ALS, amyotrophic lateral sclerosis**. **(A) The number of individuals with ALS from the Project MinE discovery cohort (n = 4299) carrying at least one rare variant in each gene encompassed in the respective column and row. (B) The number of individuals with ALS from the Project MinE discovery cohort carrying at least one rare variant in an established ALS gene encompassed in the respective column and row. (C) The number of individuals with ALS from the Project MinE discovery cohort carrying at least one primary rare variant in an established ALS gene encompassed in the respective column and at least one secondary rare variant in a known ALS gene in the respective row. Established ALS genes were defined as those with a definitive ALS gene-disease relationship based on review by the ClinGen ALS Gene Curation Expert Panel. All remaining genes assessed were considered ALS-associated genes.

In the Project MinE discovery cohort, individuals with ALS had the highest frequency of oligogenic carriers when one of the rare variants carried was in *NEK1* (1.84%) closely followed by *ANXA11* (1.81%) and the *C9orf72* repeat expansion (1.48%; figure 4A,B). However, *ANXA11* and *NEK1* were also the genes with the highest oligogenic carrier frequencies in controls (1.37% and 0.99%, respectively), whereas the *C9orf72* repeat expansion was only observed in one control with another rare variant in a known ALS gene, specifically *VAPB* (online supplemental figures 5 and 6).

The only genes for which oligogenic carriers with ALS but no controls were observed carrying at least one rare variant in the gene along with a rare variant in another known ALS gene were *CHMP2B*, *PFN1*, *SOD1* and *UBQLN2* (online supplemental figure 5). Oligogenic carriers with ALS with two or more rare variants in the most well-established ALS genes are displayed in figure 4B. Although these are considered very rare events, 129 carriers with ALS were observed across the Project MinE discovery cohort (2.8%). Oligogenic carriers with ALS with one or more rare variant(s) in a well-established ALS gene in addition to one or more rare variant(s) in an ALS-associated gene are displayed in figure 4C.

## Discussion

While evidence from previous case reports has suggested that there could be an oligogenic burden to developing ALS, this is the first large-scale study involving extensive discovery and replication datasets of thousands of people with ALS and controls. Across the two subsets of the Project MinE Sequencing Consortium, 6% of individuals with ALS were considered oligogenic carriers. This proportion was comparable to the 6.82% of Australian individuals with sporadic ALS and 3.8% of North American individuals with ALS that were found to be oligogenic carriers in cohorts of more modest sample size.^18 21^ In this oligogenic subgroup of people with ALS, we observed that the risk imparted from carrying rare variants in multiple known ALS genes was significantly greater than the risk associated with carrying only a single rare variant, in principle consistent with the multistep hypothesis of ALS.^19 20^ Further, our results have direct implication for genetic counselling and testing. Having shown that variants in more than one known ALS gene affect risk in approximately 6% of patients, it follows that all ALS-associated genes must be included in clinical genetic testing even when a potential pathogenic variant has already been identified. Without the use of comprehensive gene panels, complications may arise in cases of familial testing, whereby even in families for which there is a known pathogenic gene variant, the lack of knowledge regarding any additional variants contributing to disease risk may result in false reassurance in the case of a negative genetic test. Additionally, a lack of understanding regarding all potentially pathogenic variants carried by ALS patients may limit their enrolment in precision medicine-based clinical trials.

In contrast to the risk associations, our results suggested that the association between oligogenicity and clinical outcomes of ALS remains unclear and might not involve the same genes. The only association between ALS age of onset and carrying more than one rare variant in a known ALS gene was observed when considering missense variants and PTVs with an MAF<0.01, and the *C9orf72* repeat expansion (p=0.042). Yet, this association was only nominally significant, which—in addition to the absence of further oligogenic associations with ALS age of onset—results in a lack of clarity regarding the validity of this relationship. We only observed an association between singleton carriers and survival period when the *C9orf72* repeat expansion was included in the analysis, which may be explained by the *C9orf72* pathogenic repeat expansion being associated with a decreased survival period individually.^46 47^ Overall, consistent with recent reports,^11 48 49^ these results suggest the genetic architecture underlying ALS risk is decoupled from that underlying survival. Moreover, considering that every modification of risk is expected to correspond to an effect on age of onset,^16 50^ our results suggest that this relationship might only explain a limited proportion of the age of onset variability in ALS and the presence of concurring independent mechanisms with large effect is possible.

Oligogenic events involving the *C9orf72* repeat expansion were particularly frequent among the individuals with ALS (1.48%), the statistical power from which may contribute to our observations of the repeat’s contribution to clinical outcomes described above. As one of the most commonly inherited forms of ALS in Europeans,^51^ we sought to examine whether the oligogenic effect was primarily driven by this repeat expansion. Excluding *C9orf72,* our risk assessments confirmed that oligogenic events involving missense variants and PTVs, both in the presence and absence of the *ATXN2* repeat expansion, conferred significant risk to ALS. While a previous assessment of oligogenicity involving *C9orf72* suggested the repeat expansion was sufficient to cause ALS alone,^52^ our results suggested an increased risk from oligogenic events involving *C9orf72* in comparison to *C9orf72* singleton events. Oligogenic events could help explain the recent incomplete penetrance estimates of *C9orf72* expansions.^53^ Further, Ciura *et al* identified pathways by which the *C9orf72* and *ATXN2* pathogenic repeat expansions may genetically interact, suggesting an actual biological impact of oligogenicity involving *C9orf72* in ALS pathogenesis.^54^ Additional functional analyses will be required to determine how variants in multiple known ALS genes may synergistically induce pathology.

Many other known ALS genes were also more commonly observed oligogenic events carried by individuals with ALS than controls. Oligogenic events in the genes *CHMP2B*, *PFN1*, *SOD1* and *UBQLN2* were entirely unique to individuals with ALS in the discovery subset (absent in controls). Oligogenic events in the genes *DNAJC7*, *FUS*, *HNRNPA1*, *TUB4A4* and *VCP* or the *C9orf72* repeat expansion were only each observed in one control (0.055%). Of these 10 genes, 7 are established ALS genes—referring to those that have been classified as having a ‘definitive’ relationship with ALS according to the ClinGen ALS GCEP. While it could be proposed that oligogenic events involving established ALS genes are driving the observed risk associations, a large proportion of individuals with ALS carried oligogenic events involving only ALS-associated genes—referring to genes that have not been defined as having a definitive relationship with ALS according to the ClinGen ALS GCEP—or involving at least one variant in an established ALS gene and at least one variant in an ALS-associated gene (2.8%). We suspect that these oligogenic events involving variants in ALS-associated genes may encompass a large subset of cases in which only a singleton variant may not have contributed enough risk to drive disease onset. Yet how the two variants within each identified oligogenic event interact remains unknown, and it is possible that some events may represent cases of genetic modification—encompassing a variant driving disease risk in combination with a variant modifying disease presentation—as has been observed for oligogenic cases of complex neuropathy and retinal degeneration, among other diseases.^855^,^59^

In interpreting our results, it is important to consider that we could not examine oligogenicity involving pathogenic variants alone, due to their rarity and the resulting small number of individuals carrying two or more such variants. While this represents a limitation of our study—one that can only be addressed through future work with larger sample sizes—it is noteworthy that most oligogenic carriers in our sample harbour at least one variant of uncertain significance (VUS) contributing to disease risk. This underscores the importance of considering VUSs in the clinical management of ALS, suggesting that, as more gene therapies become available, carrying a VUS in an ALS-related gene should be sufficient for consideration of genetic treatment.

Collectively, our results reveal that oligogenic events contribute significant risk to ALS, both in the presence and absence of variants in the most well-established ALS genes, such as *C9orf72*. Moreover, the observed lack of influence of oligogenicity on survival of ALS supports the recent hypothesis of decoupling between mechanisms underlying the risk of ALS and its progression. Although our study represents the largest systematic analysis of oligogenicity to date, even greater sample sizes and variant effect studies are required to determine the exact consequences of carrying multiple ALS-associated variants on disease progression and outcomes. Nevertheless, our findings confirm that oligogenic events are relevant in ALS, which may be of particular importance when the variants involved have uncertain pathogenic significance or are observed in genes with probable ALS associations. The potential implications of these variants on ALS clinical correlates and molecular pathology warrant further exploration. In the age of stratified medication and gene therapy, implicating oligogenicity in a relevant proportion of ALS patients supports the need for a complete genetic profile for accurate genetic counselling and the correct choice of therapy in all ALS patients.

## Supplementary material

10.1136/jnnp-2024-335364 online supplemental file 1

## Data Availability

Data sharing not applicable as no datasets generated and/or analysed for this study.
