# Supplementary material for: Oligogenic structure of amyotrophic lateral sclerosis has genetic testing, counselling and therapeutic implications
Source: J Neurol Neurosurg Psychiatry. 2025 Feb 13;96(10):e335364. doi: 10.1136/jnnp-2024-335364 (PMC12505044; doi:10.1136/jnnp-2024-335364)
Supplement: online supplemental file 1 [file jnnp-96-10-s001.pdf]

Supplemental Table 1. Twenty-six known amyotrophic lateral sclerosis (ALS) genes interrogated for rare variants or repeat expansions in the genome sequencing datasets of the Project MinE ALS Sequencing Consortium.

Gene	Interrogated For	Year of First Association	Onset	Inheritance Pattern	Method of First Association	Protein Function	PMID of First Association
ANG	Rare SNVs	2006	Adult	AD	Candidate gene	RNA processing	16501576
ANXA11	Rare SNVs	2017	Adult	AD	RVA (familial/case-control studies)	Intracellular cargo transport	28469040
ATXN2	Repeat Expansion	2011	Adult	AD	RVA (familial/case-control studies)	RNA processing	21670397
C9orf72	Repeat Expansion	2011	Adult	AD	Linkage	Endosomal trafficking	21944778; 21944779
CCNF	Rare SNVs	2016	Adult	AD	Linkage	Ubiquitination	27080313
CHCHD10	Rare SNVs	2014	Adult	AD	RVA (familial/case-control studies)	Mitochondrial function	24934289
CHMP2B	Rare SNVs	2006	Adult	AD	Candidate gene	Cell maintenance	16807408
DAO	Rare SNVs	2010	Adult	AD	Candidate gene	Stress response	20368421
DCTN1	Rare SNVs	2003	Adult	AD	Candidate gene	Intracellular cargo transport	12627231
DNAJC7	Rare SNVs	2019	Adult	AD	RVA (familial/case-control studies)	Stress response	31768050
FIG4	Rare SNVs	2009	Adult	AD	Candidate gene	Endosomal trafficking	19118816
FUS	Rare SNVs	2009	Adult	AD/AR	Linkage	RNA processing	19251627
HNRNPA1	Rare SNVs	2013	Adult	AD	RVA (familial/case-control studies)	RNA processing	23455423
KIF5A	Rare SNVs	2018	Adult	AD	RVA (familial/case-control studies)	Cytoskeleton	29342275
MATR3	Rare SNVs	2014	Adult	AD	RVA (familial/case-control studies)	RNA processing	24686783
NEK1	Rare SNVs	2016	Adult	AD	RVA (familial/case-control studies)	Cell maintenance	26945885
OPTN	Rare SNVs	2010	Adult	AD	Linkage	Cell maintenance	20428114
PFN1	Rare SNVs	2012	Adult	AD	RVA (familial/case-control studies)	Cytoskeleton	22801503
SOD1	Rare SNVs	1993	Adult	AD	Linkage	Stress response	8446170
SQSTM1	Rare SNVs	2011	Adult	AD	Candidate gene	Cell maintenance	22084127
TARDBP	Rare SNVs	2008	Adult	AD	Linkage	RNA processing	18309045
TBK1	Rare SNVs	2015	Adult	AD	RVA (familial/case-control studies)	Cell maintenance	25700176
TUBA4A	Rare SNVs	2014	Adult	AD	RVA (familial/case-control studies)	Cytoskeleton	25374348
UBQLN2	Rare SNVs	2011	Adult	X-LD	Linkage	Cell maintenance	21857683
VAPB	Rare SNVs	2004	Adult	AD	Linkage	Stress response	15372378
VCP	Rare SNVs	2010	Adult	AD	RVA (familial/case-control studies)	Cell maintenance	21145000

Adapted from [1]. Abbreviations: AD, autosomal dominant; AR, autosomal recessive; RVA, rare variant association; SNVs, single nucleotides variants; X-LD, X-linked dominant.

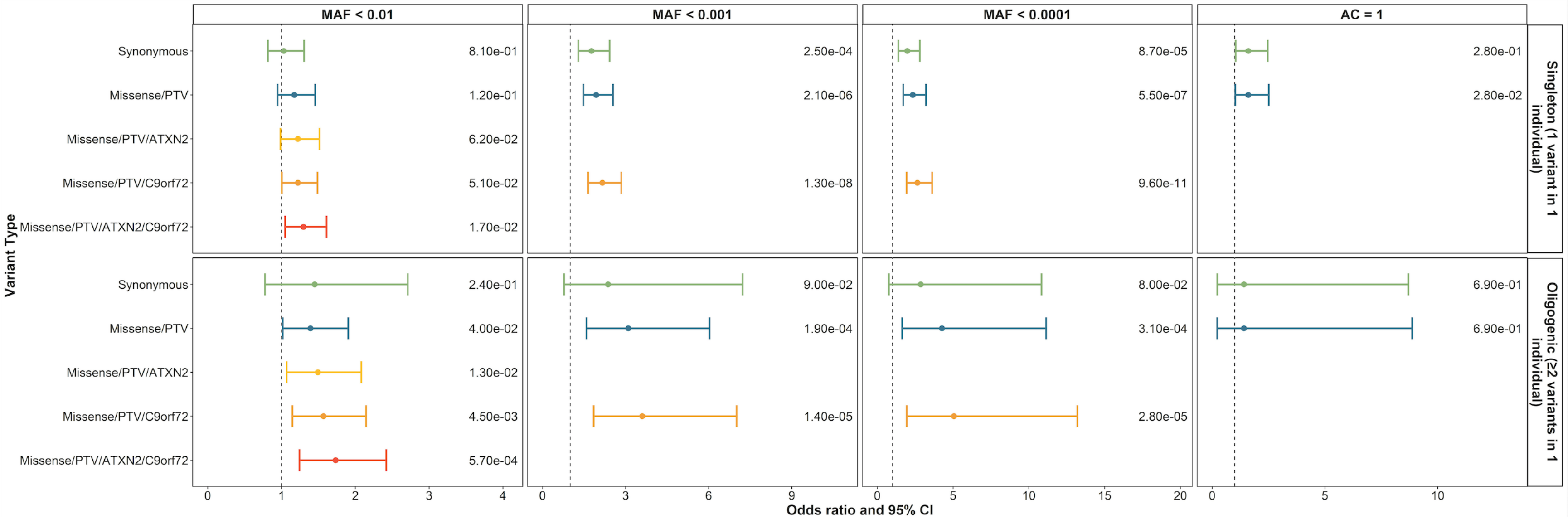

Supplemental Figure 1. Replication of singleton and oligogenic enrichment of rare variants with various MAF in known ALS genes in individuals with ALS compared to controls. Enrichment of carriers of rare variants in one known ALS gene and carriers of rare variants in two or more known ALS genes was compared to non-carriers in individuals with ALS compared to controls. Enrichment analyses were performed using logistic regression in a replication (individuals with ALS = 2057, controls = 513) subset of the Project MinE ALS sequencing consortium dataset, including sex, 10 ancestry defining principal components, and total variant count (total genetic load) as covariates. *ATXN2* and *C9orf72* refer to a pathogenic repeat expansion being identified in the respective gene using ExpansionHunter. Minor allele frequencies were obtained from the GnomAD v2.1.1 non-neurological dataset. AC = 1 refers to variants absent from the GnomAD v2.1.1 non-neurological dataset and only a single observation in the analyzed dataset. Logistic regression generated p-values are displayed on the right side of each plot. Abbreviations: AC, allele count; CI, confidence interval; MAF, minor allele frequency; PTV, protein truncating variant.

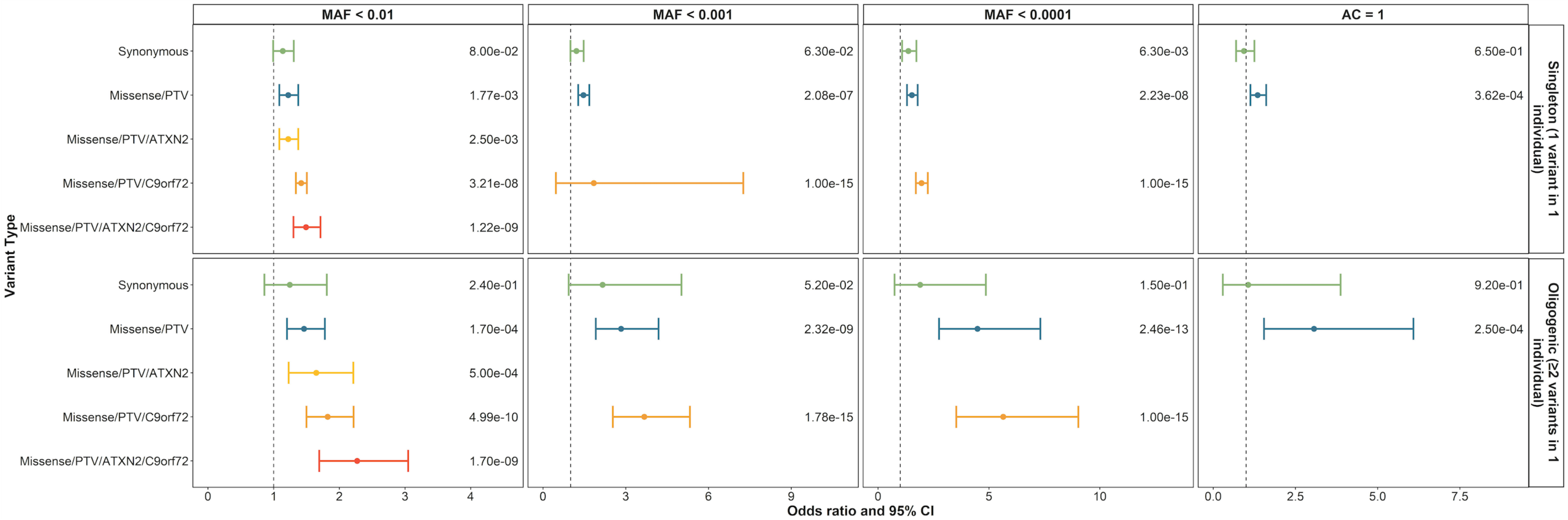

Supplemental Figure 2. Singleton and oligogenic enrichment of rare variants with various MAF in known ALS genes in individuals with ALS compared to controls. Enrichment of carriers of rare variants in one known ALS gene and carriers of rare variants in two or more known ALS genes was compared to non-carriers in individuals with ALS compared to controls. Enrichment analyses were performed using logistic regression in a discovery (individuals with ALS = 4299, controls = 1815) subset of the Project MinE ALS sequencing consortium dataset, including sex, 10 ancestry defining principal components, and total variant count (total genetic load) as covariates. *ATXN2* and *C9orf72* refer to a pathogenic repeat expansion being identified in the respective gene using ExpansionHunter. Minor allele frequencies were obtained from the GnomAD v2.1.1 non-neurological dataset. AC = 1 refers to variants absent from the GnomAD v2.1.1 non-neurological dataset and only a single observation in the analyzed dataset. Logistic regression generated p-values are displayed on the right side of each plot. Abbreviations: AC, allele count; CI, confidence interval; MAF, minor allele frequency; PTV, protein truncating variant.

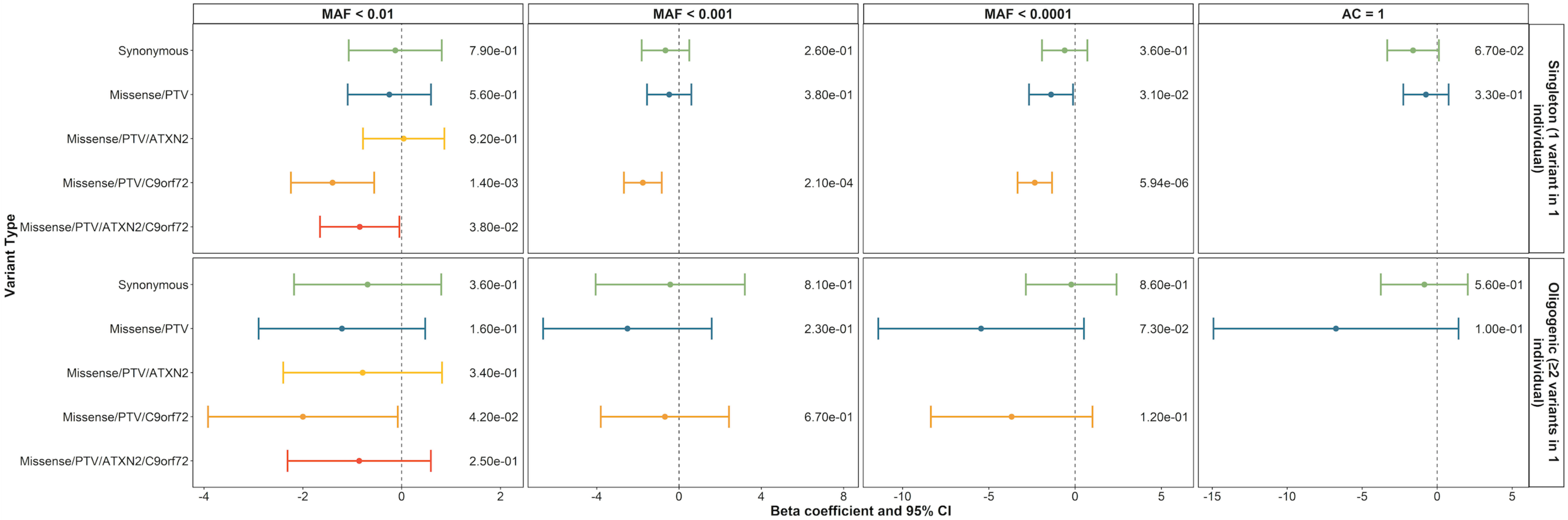

Supplemental Figure 3. Influence of singleton and oligogenic enrichment of rare variants with various MAF in known ALS genes to ALS age of onset. The influence of carrying a rare variant of various minor allele frequencies in a single known known ALS gene was compared to the influence of carrying rare variants in two or more known ALS genes on ALS age of onset in the discovery cohort of the Project MinE ALS sequencing consortium (individuals with ALS = 4299). Enrichment analyses were performed using linear regression including sex, site of onset, 10 ancestry defining principal components, and total variant count (total genetic load) as covariates. *ATXN2* and *C9orf72* refer to a pathogenic repeat expansion being identified in the respective gene using ExpansionHunter. Minor allele frequencies were obtained from the GnomAD v2.1.1 non-neurological dataset. AC = 1 refers to variants absent from the GnomAD v2.1.1 non-neurological dataset and only a single observation in the analyzed dataset. Linear regression generated p-values are displayed on the right side of each plot. Abbreviations: AC, allele count; CI, confidence interval; MAF, minor allele frequency; PTV, protein truncating variant.

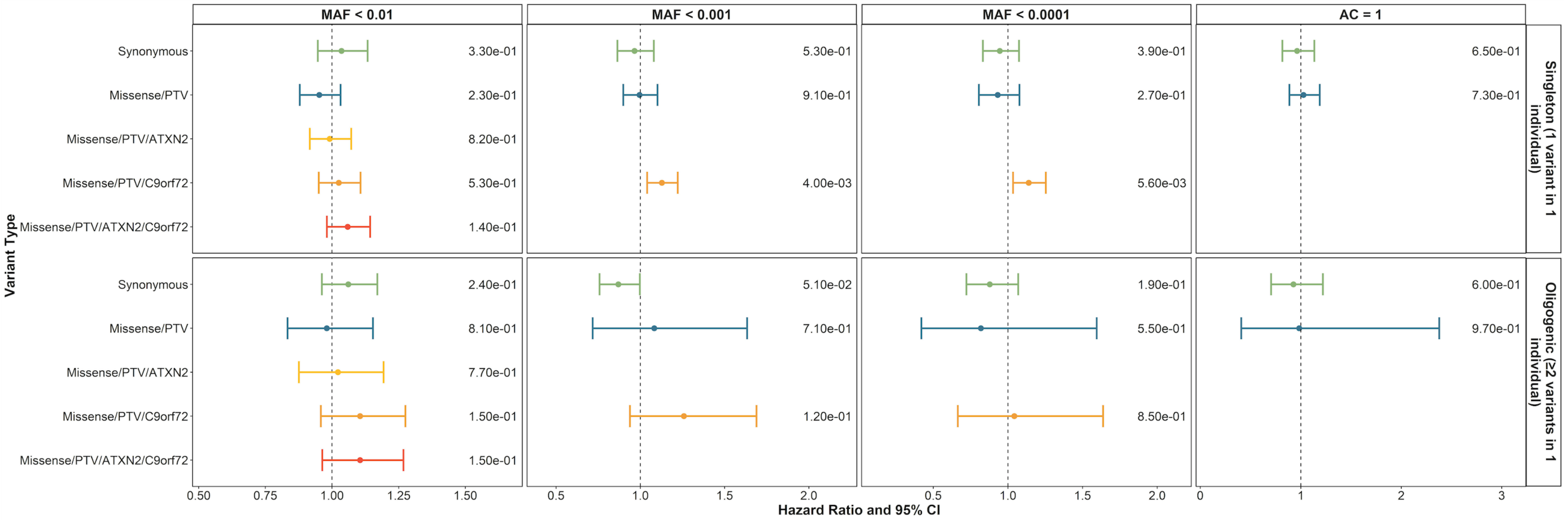

Supplemental Figure 4. Influence of singleton and oligogenic enrichment of rare variants with various MAF in known ALS genes to ALS survival period. The influence of carrying a rare variant of various minor allele frequencies in a single known ALS gene was compared to the influence of carrying rare variants in two or more known ALS genes on ALS survival period in the discovery cohort of the Project MinE ALS sequencing consortium (individuals with ALS = 4299). Enrichment analyses were performed using a Cox proportional-hazards model including sex, site of onset, 10 ancestry defining principal components, and total variant count (total genetic load) as covariates. Synonymous, missense, and protein truncating variants (PTVs) were identified in 24 known ALS genes using whole genome sequencing. *ATXN2* and *C9orf72* refer to a pathogenic repeat expansion being identified in the respective gene using ExpansionHunter. Minor allele frequencies were obtained from the GnomAD v2.1.1 non-neurological dataset. AC = 1 refers to variants absent from the GnomAD v2.1.1 non-neurological dataset and only a single observation in the analyzed dataset. Survival period was defined as years from diagnosis to death, or years from diagnosis to last follow-up, as appropriate. Cox proportional-hazards model generated p-values are displayed on the right side of each plot. Abbreviations: AC, allele count; CI, confidence interval; MAF, minor allele frequency; PTV, protein truncating variant.

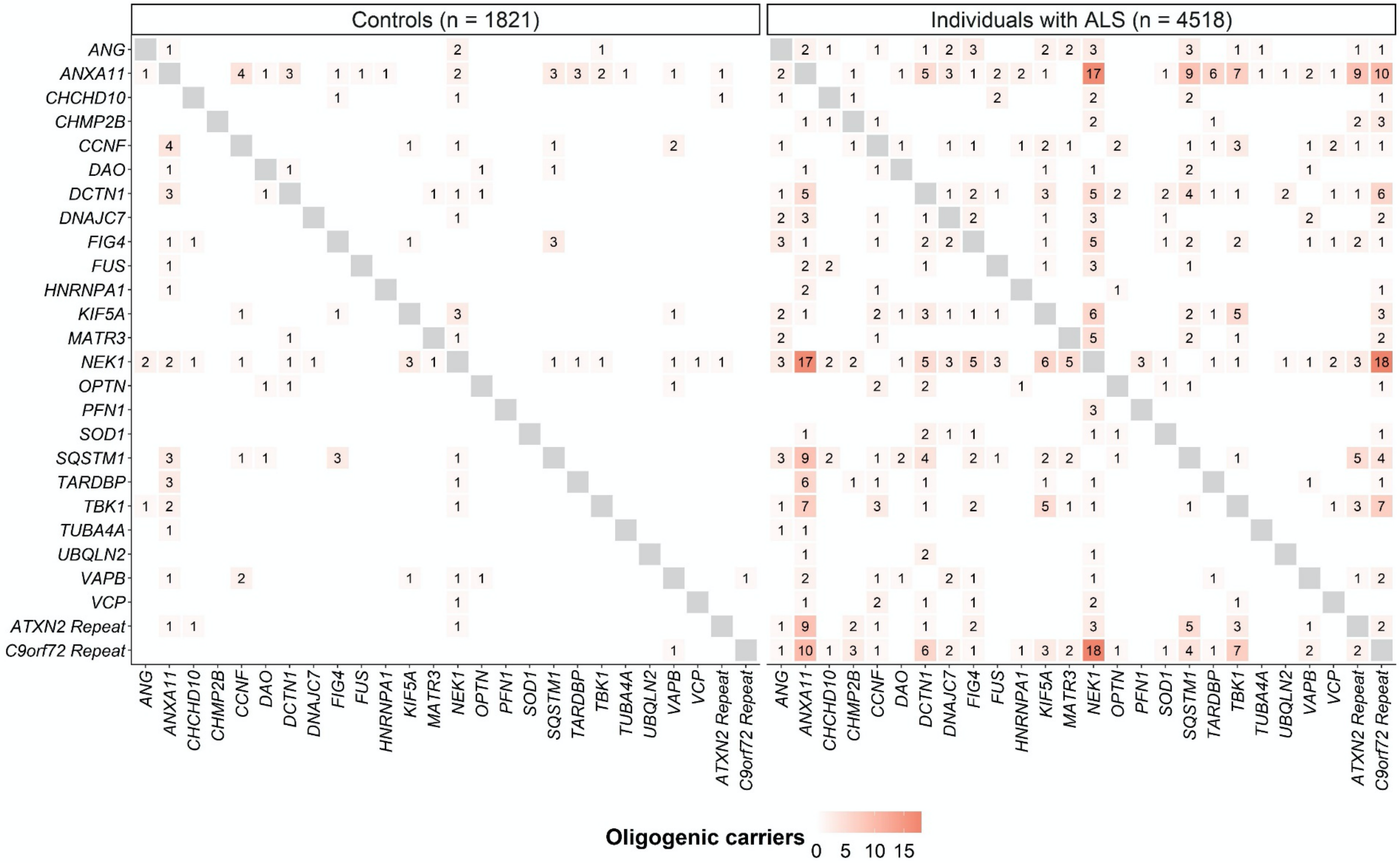

Supplemental Figure 5. Comparison of the number of individuals with ALS (n = 4,518) and controls (n = 1,821) that were oligogenic rare variant (MAF < 0.01) carriers. The gene matrices display the number of individuals with ALS and controls from the Project MinE discovery cohort carrying at least one rare variant in each gene encompassed in the respective column and row.

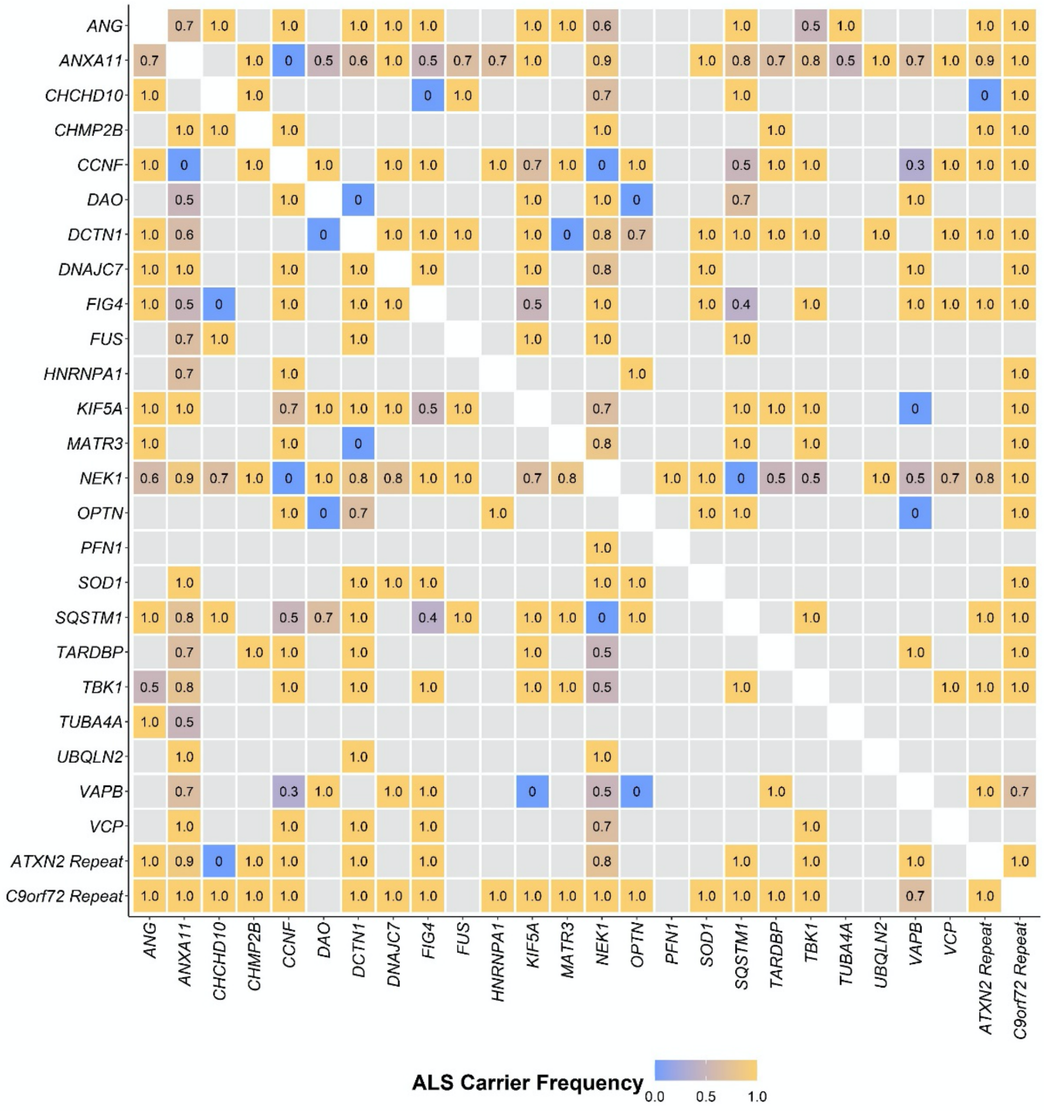

Supplemental Figure 6. Frequency of oligogenic rare variant (MAF < 0.01) carriers that had ALS. The gene matrix displays the ALS carrier frequency with at least one rare variant in each gene encompassed in the respective column and row based on the discovery cohort of the Project MinE ALS sequencing consortium (individuals with ALS = 4299, controls = 1815). ALS carrier frequency was calculated by dividing the number of individuals with ALS carrying rare variants in the specific gene combination with the total number of samples from the Project MinE discovery cohort carrying rare variants in the specific gene combination.

References:

[1] Dilliott AA, Rouleau GA, Iqbal S, Farhan SMK. Characterizing proteomic and transcriptomic features of missense variants in amyotrophic lateral sclerosis genes. medRxiv. 2022:2022.12.21.22283728. doi:10.1101/2022.12.21.22283728
